# The relationship between body dissatisfaction, lifestyle, and nutritional status among university students in Southern China

**DOI:** 10.1186/s12888-023-05215-8

**Published:** 2023-09-30

**Authors:** Ming Hao, Juan Yang, Shiliang Xu, Wenjing Yan, Hongfei Yu, Qi Wang

**Affiliations:** 1 School of Public Health and Health Management, Gannan Medical University, Ganzhou, 341000 Jiangxi China; 2 Department of Rehabilitation Medicine, Ganzhou People’s Hospital, Jiangxi, 341000 China; 3 College of Electronics and Information Engineering, Liaoning Institute of Science and Technology, Benxi, 117000 Liaoning China; 4 Key Laboratory of Prevention and Treatment of Cardiovascular and Cerebrovascular Diseases, Ministry of Education, Gannan Medical University, Ganzhou , Jiangxi, 341000 China

**Keywords:** Body dissatisfaction, Lifestyle, Nutritional status, University students, China

## Abstract

**Background:**

In recent years, obesity in early adulthood has become an urgent global public health concern. Body dissatisfaction may have adverse effects on lifestyle habits, leading to obesity. However, research on nutritional status and body dissatisfaction among Chinese young adults is still insufficient. Therefore, this study aimed to analyze the relationship between body dissatisfaction, dietary habits, physical activity, and nutritional status among university students. In addition, we explored the feasibility of improving university students’ nutritional status by improving the levels of body dissatisfaction.

**Methods:**

This study was conducted in Ganzhou City, Jiangxi Province, China, at a randomly selected university. All 1900 undergraduate students volunteered to participate and signed the consent form. Students were required to completed anthropometric measurements and three questionnaires, which included the Physical Activity Rating Scale-3 (PARS-3), Chinese version of the Dutch Dietary Behavior Questionnaire (C-DEBQ), and Body Dissatisfaction. Of these, 1714 students (age: 18–24 years; men: 933, women: 781) with complete and valid data were included.

**Results:**

Higher obesity levels were observed in men compared to women (*p*<0.01). Meanwhile, body dissatisfaction was higher in women compared to men (*p*<0.01). Overeating and insufficient physical activity were more problematic in women compared to in men (*p*<0.01). Multiple regression analyses were conducted separately, with BMI and body dissatisfaction as the dependent variables. Body dissatisfaction (β=0.72, *p*<0.01), muscle mass (β=0.33, *p*<0.01), emotional eating score (β=0.05, *p*<0.01), sex (β=-0.05, *p*<0.05) and physical activity (β=-0.04, *p*<0.05) score were significant predictors of obesity. Furthermore, Muscle mass (β=0.61, *p*<0.01), sex (β=0.54, *p*<0.01), restrained eating score (β=0.25, *p*<0.01), physical activity score (β=-0.20, *p*<0.01) and emotional eating score (β=0.08, *p*<0.01) were significant predictors of body dissatisfaction.

**Conclusion:**

The data presented in this study highlight the impact of university students’ body dissatisfaction in China on physical activity deficiency and overeating, discovering that reducing body dissatisfaction has great potential for preventing obesity.

## Introduction

Owing to economic development, obesity has become animportant public health issue. According to the World Health Organization (WHO), as of 2016, more than 2.5 billion adults were overweight or obese [1]. Simultaneously, the problem of obesity is no longer unique to developed countries, and the recent rapid increase of obesity in certain low- and middle-income developing countries has become a growing concern [2, 3]. Interestingly, obesity rates in developed countries stabilized while rates in developing countries continue to rise [4]. Obesity is a risk factor for lifestyle diseases, such as hypertension, coronary heart disease, and stroke [5]. Although the symptoms usually appear in middle-aged individuals, they develop gradually and may appear in adolescence [6].

Unhealthy eating behaviors and insufficient physical activity are a major cause of obesity [7, 8]. Many studies have reported unhealthy eating behaviors among young people. A study on 13,917 U.S. high school students revealed that 16.6% had engaged in eating disorder behaviors [9]. A study on Chinese university students revealed that 2.5% exhibited risky eating attitudes [10]. A pooled analysis based on 358 studies from various countries worldwide, which covered 1.9 million respondents, showed that 25% of adults had insufficient physical activity [11]. Meanwhile, a study revealed that only 10% of Chinese adults engaged in regular physical activity [12]. Insufficient physical activity has been associated with obesity [13]. Hence, improving eating behaviors and insufficient exercise of young adults is particularly useful when attempting to prevent obesity.

Body image is the way people feel regarding the size and shape of their body [14]. Body dissatisfaction occurs when there is a discrepancy between a person's actual and ideal body size [15]. Pioneering research has shown that body dissatisfaction could affect eating and exercise habits [7, 16]. Due to increasing obesity, increasing research has focused on the relationship between obesity and body dissatisfaction [17].

University life is a transitional stage for students between adolescence and adulthood [18]. Without parental supervision, college students may find it difficult to maintain healthy eating and exercise habits [7, 18]. Dissatisfaction with one's body is common in this population [19–21]. This may increase the likelihood of college students choosing extreme ways to change their appearance [20, 21]. Since a healthy lifestyle during university years can positively influence a future healthy lifestyle, it is crucial to foster its development among university students [22]. However, research on nutritional status and body dissatisfaction among Chinese young adults is still insufficient. Therefore, this study aimed to analyze the relationship between body dissatisfaction, eating behavior, physical activity, and nutritional status and explore the feasibility of improving university students’ nutritional status by improving their levels of body dissatisfaction.

## Materials and methods

### Study participants

A cross-sectional study was designed for university students. This study was conducted in Ganzhou City, Jiangxi Province, China, at a randomly selected comprehensive university. Participant recruitment information was disseminated on campus through posters placed in the dormitory building and leaflets distributed in the study rooms. Between June 2021 and February 2022, a total of 1900 undergraduate students volunteered to participate and signed the consent form. Physical measurements were taken and the questionnaires were administered to the participants. Of these, 1714 students (age: 18–24 years; men: 933, women: 781) with complete and valid data were included.

### Body measurements

Height was measured using a height tape with an accuracy of 0.1 cm. Body weight (0.1 kg), muscle mass (1 kg), and body fat (%) were measured using a body composition analyzer (BC754, Tanita). Body mass index (BMI, kg/m2) was calculated using height and weight; the BMI categories were underweight (BMI < 18.5), normal (18.5 ≤ BMI < 25), and overweight (BMI ≥ 25) [23].

### Body dissatisfaction

Using a questionnaire, the ideal weight of university students was investigated in 0.1 kg increments. The ideal BMI was calculated based on their actual height and ideal weight, and body dissatisfaction was calculated by combining the actual and ideal BMI values. The difference between the actual and ideal BMI values was considered as the body dissatisfaction score [24]. This method has been previously used to measure body dissatisfaction among Chinese university students [6].

### Eating behavior

The Chinese Version of the Dutch Eating Behavior Questionnaire [25] was used to evaluate the level of overeating. The 33-item scale was divided into three categories: restrained, emotional, and external eating. Thirteen questions were set for emotional eating, such as “Do you have the desire to eat when you are irritated?”; ten questions were set for external eating, such as “Do you eat more than usual when you see others eating?”; and ten questions were set for restrained eating, such as “Do you find it hard to resist eating delicious foods?”. Items were rate on 5-point scale, and higher total scores on each subscale indicated more frequent eating behaviors. In addition, higher scores indicated higher levels of overeating. For this dataset, internal reliability coefficients (Cronbach’s α) were 0.88 for the restrained subscale, 0.84 for the emotional eating scale, and 0.82 for the external eating scale.

### Physical activity level

The Physical Activity Rating Scale-3 (PARS-3), a three-question scale, includes three broad dimensions: exercise time (ET), exercise intensity (EI) and frequency (EF), a 5-item self-report scale covering duration, intensity, and frequency [26]. Rating of each item on a scale of 1 to 5 and the total score for physical activity (i.e., exercise volume) were computed using the following equation: EI × (ET-1) × EF, which represented the score of exercise intensity that ranged from 0 to 100 and was further classified into three levels according to the score: high (> 43), moderate (20–42), and low (0–19) exercise intensity. The scale has been widely used with good reliability and validity [26]. In addition, the Cronbach’s a of PARS-3 in the current study was 0.81.

### Patient and public involvement

The public was not involved in the study design, conduct of the study, or plans to disseminate the results to study participants.

### Statistical analyses

Independent samples t-tests were used to verify sex differences in BMI, body fat percentage, muscle mass, level of body dissatisfaction and physical activity, and eating behavior scores. Pearson’s chi-squared test was used to compare the sex differences in BMI categories and physical activity. Tukey’s test was used to compare the total eating behavior scores among the different BMI groups. Multiple regression analyses were conducted separately, with BMI and body dissatisfaction as the dependent variables. When BMI was used as the dependent variable, sex (men: 0; women: 1), cost of living, muscle mass, three categories of eating behavior scores, total eating behavior and physical activity scores, and body dissatisfaction were used as the predictor variables. When body dissatisfaction was used as the dependent variable, sex (men: 0; women: 1), cost of living, muscle mass, three categories of eating behavior scores, and total eating behavior and physical activity scores were used as predictor variables. Variables were selected according to stepwise increase and decrease methods, and threshold *P*-values were calculated using likelihood ratio tests and set at 0.20. Statistical significance was set a *p* < 0.05. JMP ver.16.01J (SAS Institute, Inc, Cary, NC) was used for all statistical analyses.

### Sample size estimation

The sample size for the study was determined using the G*Power calculator 3.1.9.7 (Franz Faul et al., Universität Kiel, Germany, http://www.gpower.hhu.de/). Considering an α = 0.05, 1-β = 0.90, the number of tested predictors = 7 (BMI, body dissatisfaction, physical activity score, restrained eating score, emotional eating score, external eating score and muscle mass), the number of covariates = 2 (age and sex), we calculated the sample size to be 71, 153, 1099, respectively if the effect size f^2^ equaled to 0.35 (large), 0.15 (medium) and 0.02 (small). Furtherly, a 20% dropout rate was assumed, and the total number was estimated as 85—1318. To make sure the power, we increased the sample size to 1900, and the actual valid sample size was 1714, which was much larger than the estimated size even if given a small effect size f^2^.

## Results

### Participants’ general characteristics

Table 1 shows the participants’ characteristics. The average age of the study subjects is 19.8 years old. The average BMI for men is 22.2, while for women it is 21.2. The average muscle mass for men is 51.1 kg, while for women it is 36.3 kg. Both BMI and muscle mass were higher in men compared to in women (*p* < 0.01). The average level of body dissatisfaction was significantly higher in women (*p* < 0.01). Overweight and obesity rates reached 26% and 14% for men and women, respectively.

**Table 1:** Participants’ characteristics (*n*=1714)

	Mean ± SD or n (%)			*p*^†^
	Men (*n* = 933)	Women (*n* = 781)	Total (*n* = 1714)	
age	19.8 ± 2.0	19.7 ± 1.7	19.8 ± 1.9	> 0.05
BMI (kg/m^2^)	22.2 ± 3.7	21.2 ± 3.0	21.8 ± 3.4	< 0.01
Muscle mass (kg)	51.1 ± 7.7	36.3 ± 4.5	44.4 ± 9.8	< 0.01
Body dissatisfaction(kg/m^2^)	1.0 ± 3.2	2.1 ± 2.3	1.5 ± 2.9	< 0.01
BMI category				
Underweight	87 (9)	83 (11)	170 (10)	< 0.01
Normal	601 (65)	588 (75)	1189 (69)	
Overweight and Obese	245 (26)	110 (14)	355 (21)	

*BMI* Body mass index

^†^The significance of differences between male and female students was determined by t-test (BMI, muscle mass, and body dissatisfaction) or Pearson’s analyses (BMI category)

### Eating behaviors

**Fig. 1:**
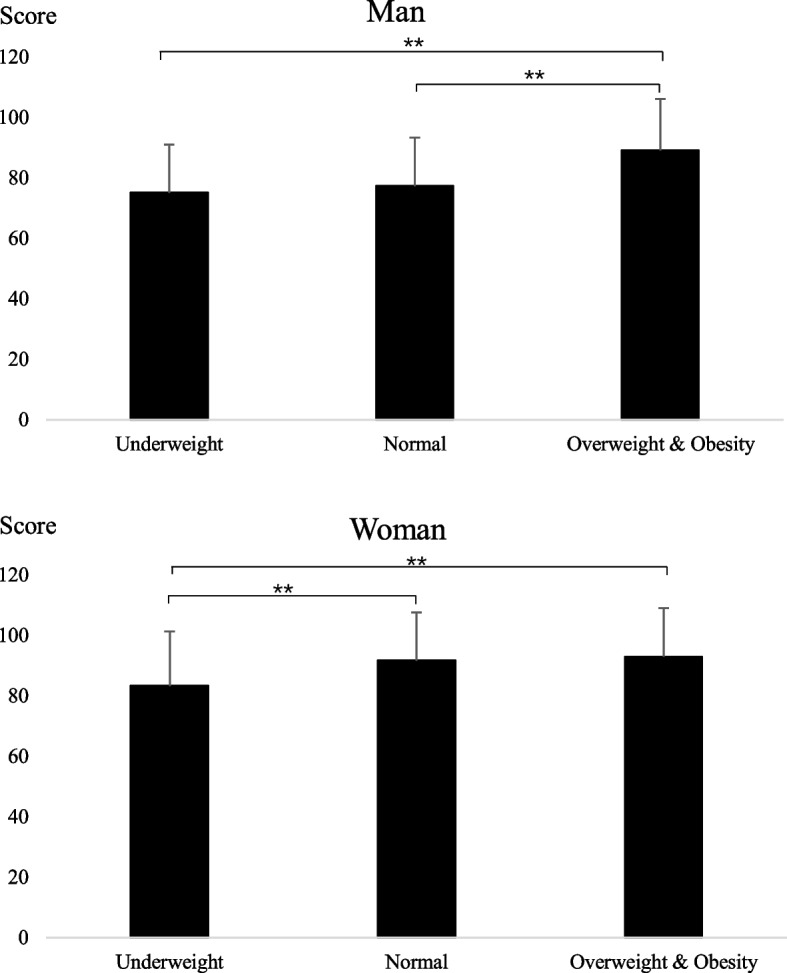
Total eating behavior score of university students in the different BMI category groups. ** Tukey-test, *p*<0.01

The three categories of eating behavior scores and total scores were all higher for women compared to men (*p* < 0.01) (Table 2). The total eating behavior score of the overweight and obese man groups was higher than that in the normal and underweight groups (*p* < 0.01) (Fig. 1). The total eating behavior scores of the overweight and obese women and normal group were higher than that of those in the underweight group (*p* < 0.01) (Fig. 1).

**Table 2:** Sex difference in the eating behaviors and physical activity among university students (*n*=1714)

	Mean ± SD or n (%)			*p*^†^
	Men (*n* = 933)	Women (*n* = 781)	Total (*n* = 1714)	
Eating behavior				
Emotional eating score	24.1 ± 10.1	26.3 ± 10.0	25.1 ± 10.1	< 0.01
External eating score	31.0 ± 8.1	35.8 ± 7.1	33.2 ± 8.0	< 0.01
Restrained eating score	24.6 ± 8.1	28.5 ± 7.5	26.4 ± 8.1	< 0.01
Total eating behaviors score	79.6 ± 19.6	90.5 ± 18.2	84.6 ± 19.7	< 0.01
Physical activity				
Physical activity score	21.8 ± 21.2	11.4 ± 14.8	17.1 ± 19.2	< 0.01
Physical activity category				
Low exercise	563 (60)	659 (84)	1222 (71)	< 0.01
Medium exercise	203 (18)	75 (10)	278 (16)	
High exercise	167 (22)	47 (6)	214 (13)	

^†^The significance of differences between male and female students was determined by t-test (emotional eating score, external eating score, restrained eating score, total eating behaviors score, and physical activity score) or Pearson’s analyses (physical activity category)

### Physical activity level

Table 2 shows the university students’ physical activity levels. The mean physical activity scores of men and women were 21.8 and 11.4 respectively. Men's mean physical activity scores are close to twice that of women. The mean physical activity scores were significantly higher in men compared to in women (*p* < 0.05). In total, 60% and 84% of men and women were categorized into the low exercise level group, respectively.

### Factors that influenced nutritional status

Table 3 shows the factors influencing obesity among university students. Body dissatisfaction (β = 0.72, *p* < 0.01), muscle mass (β = 0.33, *p* < 0.01), emotional eating score (β = 0.05, *p* < 0.01), sex (β = -0.05, *p* < 0.05) and physical activity (β = -0.04, *p* < 0.05) score were significant predictors of obesity.

**Table 3:** The impact of body dissatisfaction, muscle mass, physical activity, eating behaviors and sex on BMI

	β	*t*	VIF	*p*
Body dissatisfaction	0.72	46.73	1.61	< 0.01
Muscle mass (kg)	0.33	15.67	3.06	< 0.01
Emotional eating score	0.05	3.45	1.16	< 0.01
Sex (Man: 0; Woman: 1)	-0.05	-2.32	3.15	< 0.05
Physical activity score	-0.04	-2.02	1.31	< 0.05

*VIF* Variance inflation factor. R^2^: 0.75; *p* < 0.01; Root Mean Square Error (RMES): 1.71

### Factors that influenced body dissatisfaction

Table 4 shows the factors influencing the level of body dissatisfaction. Muscle mass (β=0.61, *p*<0.01), sex (β=0.54, *p*<0.01), restrained eating score (β=0.25, *p*<0.01), physical activity score (β=-0.20, *p*<0.01) and emotional eating score (β=0.08, *p*<0.01) were significant predictors.

**Table 4:** The impact of muscle mass, physical activity, eating behaviors, and sex on body dissatisfaction

	β	*t*	VIF	*P*
Muscle mass (kg)	0.61	20.43	2.45	< 0.01
Sex (Man: 0; Woman: 1)	0.54	17.09	2.68	< 0.01
Restrained eating score	0.25	11.83	1.23	< 0.01
Physical activity score	-0.20	-10.27	1.09	< 0.01
Emotional eating score	0.08	3.51	1.29	< 0.01

*VIF* Variance inflation factor. R^2^: 0.38; *p* < 0.01; Root Mean Square Error (RMES): 2.28

## Discussion

In China, obesity rates were generally higher in men than in women [27]. Our results showed that the overweight and obesity rate for male university students was 26%, which was much higher than the 14% for female students (Table 1). Hence, our findings supported those of the previous study. With the development of media, being thin is a deeply rooted standard of beauty among women [28]. The women believe that slender bodies are more attractive [29]. In addition, women with slim bodies are likely to have access to more resources in society, such as easier access to jobs that require a slim shape [30]. For women, the pursuit of body shape is now more of an aesthetic need rather than just regarding health [31].

Studies revealed that women had higher levels of body dissatisfaction compared to men [6, 32, 33]. Similarly, our results showed that women exhibited higher levels of body dissatisfaction compared to men (Tables 1 and 4), supporting the findings of previous studies. Research has found that women were more susceptible to media influence and had a greater desire to have a slimmer body [6, 28]. However, unlike women, men had a greater desire to improve muscle strength [34]. Therefore, the difference in an ideal body size between men and women was an important reason for sex differences in the levels of body dissatisfaction. Simultaneously, in Chinese culture, obesity in men is usually considered a symbol of affluence [35]. Hence, the reasons for differences in body dissatisfaction between men and women were related to the women’s psychological characteristics and culture.

Overeating is a problem in overweight and obese individuals [36]. Similar results were found among the male students in the present study (Fig. 1). However, there was no difference in the total eating behavior scores between female students in the normal weight group and those in the overweight and obese group (Fig. 1). Overeating is also a common problem among female university students. A study on 3714 women in the U.S. found that 18% had binge eating problems, and more than 10% engaged in binge eating behavior at least once a week [37]. Driven by body dissatisfaction, women are more likely to take action to improve their body image, even with extreme behaviors, such as dieting, compared to men [38]. Our results showed that female university students had higher scores compared to male students on all eating behaviors (Table 2), which supported findings of previous studies. Additional research revealed that some individuals used binge eating as a counter mechanism for short-term relief of distressing emotions [39]. The biological differences between women and men may be reflected in emotions and behavior, with women likely to be more emotional in the face of challenging and uncertain situations [40]. Hence, overeating is more likely in women compared to men.

Our results also showed that body dissatisfaction was a significant influencing factor of obesity (Table 3). Several studies demonstrated a positive association between body dissatisfaction and obesity [41–43]. Obese people were more likely to be dissatisfied with their body shape, exhibited a greater desire to have a slimmer body, and thus, had higher levels of body dissatisfaction compared to non-obese healthy people [41, 42]. However, recent research found that the relationship between body dissatisfaction and obesity was bidirectional [6]. Body dissatisfaction also had a significant influence on obesity (Table 3). At the same time, body dissatisfaction is prevalent in non-obese girls [6]. Thus, high body dissatisfaction may lead to more obesity.

Research also revealed that the presence of overeating in normal-weight young people, particularly emotional binge eating, could contribute to weight gain and subsequent obesity [44]. Our results showed that higher emotional eating scores were associated with higher levels of obesity in university students (Table 3), which supported previous findings. Notably, we also found that increased levels of body dissatisfaction could lead to increased emotional eating (Table 4). Thus, our findings supported those of previous studies [44, 45]. Therefore, improving body dissatisfaction played an important role in improving university students’ eating habits.

Exercise is important in obesity management. It is thought that an increase in exercise will burn more energy, leading to weight loss. The WHO suggested that the potential risk of chronic disease can be reduced through moderate and vigorous physical activity [46]. In this study, over 60% and 80% of male and female students exhibited low or no physical activity levels, respectively (Table 2). Another survey among university students in Guangzhou, China, found that 73% regularly engaged in low exercise or did not exercise [26]. We found that the lower the level of exercise, the higher the BMI level of the university students (Table 3). Lack of physical exercise habits may be one of the reasons for obesity among southern university students.

Body dissatisfaction may reduce exercise levels among university students (Table 4). Some studies showed that body dissatisfaction may lead to reluctance or intentional avoidance of participation in physical activity. A study found that people with high body dissatisfaction were more likely to feel embarrassed in sports as they were not confident in their body image [19]. In addition, people with high body dissatisfaction avoided activities that involved motor skills due to the fear of being perceived as unattractive [47]. Some studies also revealed that participating in physical activity to change body image was less sustainable than participating in physical activity for health [6, 19]. Therefore, reducing body dissatisfaction and developing health awareness is important to improve physical activity levels and prevent obesity in adolescents.

A previous study found the impact of body dissatisfaction on physical activity and dietary behavior [6]. This study further discovered the possibility of improving body dissatisfaction in reducing the risk of obesity among university students. At the same time, we have proposed new directions for the development of obesity prevention methods for university students. In future research, it is necessary to verify the effectiveness of reducing body dissatisfaction levels through body image education in preventing and improving the obesity status of Chinese university students.

### Strengths and limitations

Our study explores the relationship between body dissatisfaction, lifestyle, and nutritional in a representative university student population and proves that improving body dissatisfaction has the potential of reducing obesity risk among university students. This study has several limitations. This study was conducted with 1714 participants, which means that the results of this study may lack universality. And included only Chinese university students. It is therefore difficult to generalize to other countries and age groups.

## Conclusion

Obesity, overeating, and insufficient physical activity are common problems among university students in southern China. In our study, obesity levels were higher in men compared to in women. However, overeating, and insufficient physical activity was more prominent in women. The data presented here highlight the impact of university students’ body dissatisfaction in China on physical activity deficiency and overeating, discovering that reducing body dissatisfaction has great potential for preventing obesity.

## Data Availability

The datasets used and analyzed in the current study are available from the corresponding author on reasonable request.
